# Using artificial intelligence reading label system in diabetic retinopathy grading training of junior ophthalmology residents and medical students

**DOI:** 10.1186/s12909-022-03272-3

**Published:** 2022-04-09

**Authors:** Ruoan Han, Weihong Yu, Huan Chen, Youxin Chen

**Affiliations:** Department of Ophthalmology, Peking Union Medical College Hospital, Key Laboratory of Ocular Fundus Diseases, Chinese Academy of Medical Sciences, Beijing, 100730 China

**Keywords:** Diabetic retinopathy, Artificial intelligence, Grading training

## Abstract

**Purpose:**

Evaluate the efficiency of using an artificial intelligence reading label system in the diabetic retinopathy grading training of junior ophthalmology resident doctors and medical students.

**Methods:**

Loading 520 diabetic retinopathy patients’ colour fundus images into the artificial intelligence reading label system. Thirteen participants, including six junior ophthalmology residents and seven medical students, read the images randomly for eight rounds. They evaluated the grading of images and labeled the typical lesions. The sensitivity, specificity, and kappa scores were determined by comparison with the participants’ results and diagnosis gold standards.

**Results:**

Through eight rounds of reading, the average kappa score was elevated from 0.67 to 0.81. The average kappa score for rounds 1 to 4 was 0.77, and the average kappa score for rounds 5 to 8 was 0.81. The participants were divided into two groups. The participants in Group 1 were junior ophthalmology resident doctors, and the participants in Group 2 were medical students. The average kappa score of Group 1 was elevated from 0.71 to 0.76. The average kappa score of Group 2 was elevated from 0.63 to 0.84.

**Conclusion:**

The artificial intelligence reading label system is a valuable tool for training resident doctors and medical students in performing diabetic retinopathy grading.

## Summary statement

This article evaluates the efficiency of using an artificial intelligence reading label system in the diabetic retinopathy grading training of junior ophthalmology resident doctors and medical students. Through reading training, the kappa score of the DR grading was elevated. It showed that the artificial intelligence reading label system was a valuable tool in training resident doctors and medical students in doing diabetic retinopathy grading.

## Introduction

Diabetic retinopathy (DR) is the most common microvascular complication of diabetes and the leading cause of irreversible vision loss in working-age adults [1]. The prevalence of diabetes in China is estimated to be around 10–11% [2–4], Thus, China has the largest population of diabetes in the world, creating a high burden of DR. [5] Early diagnosis and treatment of DR can cause timely medical intervention, thus preventing progression of the disease and avoiding the occurrence of severe visual impairment [6, 7]. Therefore, it is crucial to accurately screen and grade the disease. According to the White Paper on Eye Health in China, there are about 44,800 ophthalmologists in China [8]. Among these, qualified specialists in fundus diseases are in a severe short supply. An effective DR screening programme should ensure that screeners and graders are systematically trained and qualified to read DR photos; the duration of this training process usually takes several months. For example, in the case of the UK Gloucestershire Retinal Education Group DR screening project, the total course duration was 40 weeks [9]. If certain methods can reduce the time required for training, it will significantly improve the efficiency of DR screening and will be beneficial for DR prevention and control.

In recent years, because of the rapid development of artificial intelligence (AI) techniques, AI techniques based on machine learning play a significant role in DR screening, which acquires high sensitivity and specificity through the learning of a large number of fundus photo training data sets [10–18]. But the fundus photo training data sets needed manual annotation by qualified specialists, and the AI reading results also needed to be confirmed by retina experts. Thus, to train junior ophthalmologists in DR reading and AI data set annotation, DR reading training is vital for ophthalmology residency training. The purpose of this study is to evaluate the efficiency of using an artificial intelligence reading label system in the diabetic retinopathy grading training of junior ophthalmology resident doctors and medical students.

## Methods

### Reading methods

A total of 520 fundus photographs centered on the macular region were included in this study. Photographs were randomly divided into 8 groups, with 65 images for each group. The severity of diabetic retinopathy was graded based on the international clinical diabetic retinopathy severity scale [19]. Photographs of no DR, mild non-proliferative DR (NPDR), moderate NPDR, severe NPDR and proliferative DR (PDR) were included in each group. Three senior consultants made the diagnosis gold standard for each image. Participants were randomly recruited from all first-year ophthalmology residents and medical students entering clinical studies who were interested in this training. Thirteen junior ophthalmology residents and medical students participated in the training. Six of them were first-year residents of the ophthalmology residency training programme at Peking Union Medical College Hospital (PUMCH). Seven of them were medical students at Peking Union Medical College (PUMC). Thirteen participants performed DR reading using the AI reading label system, made DR grading, and labelled the classic lesions of each image. Reading training was performed for 8 rounds with 65 images per round. After each round’s labelling, the participants were gathered to study the diagnosis gold standard. Each round lasted for 1 week, and the whole process lasted for 8 weeks. The sensitivity and specificity according to the diagnosis glod standard were summarized after each round.

### Grading methods

Fundus photographs were divided into 5 levels according to the DR severity degrees. No DR, mild NPDR, moderate NPDR, severe NPDR, or PDR were labelled as degrees 0, 1, 2, 3, or 4, respectively. Degree 0 is defined as ‘without DR’ and degrees 1 to 4 are defined as ‘with DR’. Degrees 0 and 1 are defined as ‘non-referral DR’, while degrees 2 to 4 are defined as ‘referral DR’. Degrees 0 to 2 are defined as ‘non-severe DR’, while degrees 3 and 4 are defined as ‘severe DR’.

### Introduction to the Reading label system

The reading label system was originally developed for manual grading and annotation in training the AI deep learning model. It was a web-based annotation system and provided adaptively enhanced versions of the original images for reference. The readers logged in with their accounts, and the system loaded a certain number of images randomly. After reading the photos and marking the main abnormal lesions, the readers chose a grade, and the system automatically compared the results with the gold standards to calculate sensitivity and specificity.

### Statistical methods

Diagnosis results were collected according to the diagnosis golden gold standard and analyzed statistically using SPSS 25 (IBM, NY, USA). Three diagnosis classifications were set as with/without DR, referral/non-referral DR, and severe/non-severe DR. We calculated the sensitivity and specificity of each classification. Sensitivity was calculated as the number of correctly diagnosed positive examples divided by the total number of positive examples. The specificity was calculated as the number of correctly diagnosed negative examples divided by the total number of negative examples. The harmonic mean of the sensitivity and specificity of each classification was calculated. The kappa score was calculated by combining the harmonic means of the three classifications. Kappa scores of 0.61 to 0.80 were determined to be of significant consistency, while kappa scores above 0.80 were determined to be highly consistent. The discrepancy in kappa scores before and after training was compared to evaluate the effect of DR reading training.

## Results

### Training results for all the participants

**Fig. 1:**
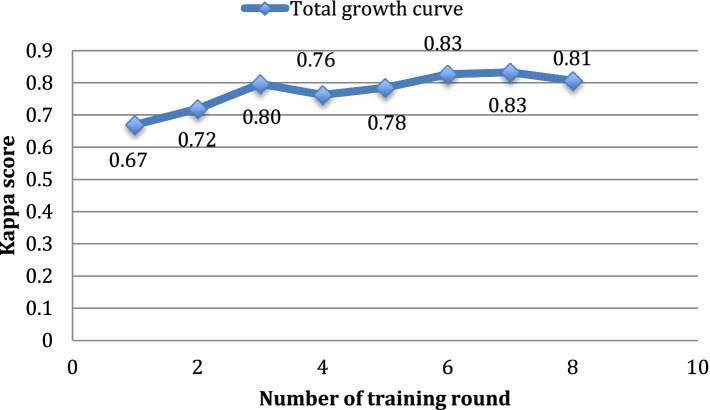
Growth curve of the average kappa scores for each reading round for the 13 reading training participants. The abscissa is the number of training rounds, and the ordinate is the Kappa score

Thirteen participants were randomly recruited from all first-year ophthalmology residents and medical students entering clinical studies, including three men and ten women. The average age of the participants was 25.54 ± 2.96 years. In the DR reading training, the average harmonic means of each diagnosis classification and the average kappa scores are shown in Table 1. Through the eight rounds of reading, the average kappa score was elevated from 0.67 to 0.81. The average kappa score of the first 4 rounds was 0.77, which means significant consistency. The average kappa score of the latter 4 rounds was elevated to 0.81, which signifies highly consistent. There has been an escalating trend in diagnostic accuracy. The growth curve of reading training is shown in Fig. 1.

**Table 1:** Average harmonic mean and average kappa score of each round

		Number of rounds							
		1	2	3	4	5	6	7	8
Average harmonic mean	With/without DR	0.55	0.64	0.71	0.65	0.79	0.74	0.82	0.73
	Referral/non-referral DR	0.76	0.79	0.84	0.75	0.85	0.86	0.85	0.81
	Severe/non- severe DR	0.75	0.76	0.88	0.86	0.79	0.89	0.84	0.85
Average value of kappa score		0.67	0.72	0.80	0.76	0.78	0.83	0.83	0.81

*Abbreviations*: *DR* Diabetic retinopathy

The harmonic mean of with/without DR was elevated from 0.55 to 0.73, and the harmonic mean of referral/non-referral DR was elevated from 0.76 to 0.81. The harmonic mean of severe/non-severe DR was elevated from 0.75 to 0.85.

### Training results for each group

**Fig. 2:**
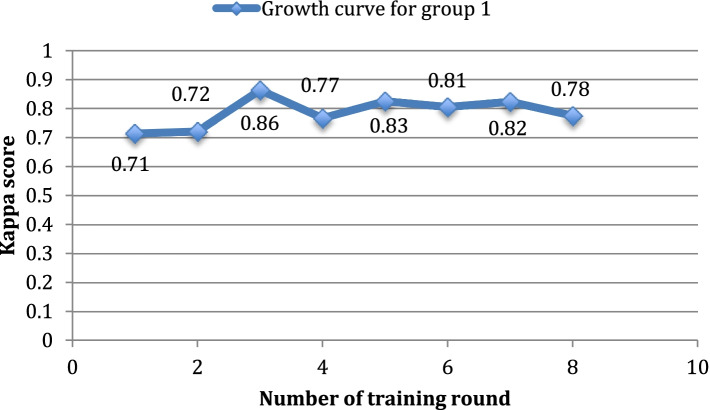
Growth curve of the average kappa scores for Group 1 (junior ophthalmology residents). The abscissa is the number of training rounds, and the ordinate is the Kappa score

**Fig. 3:**
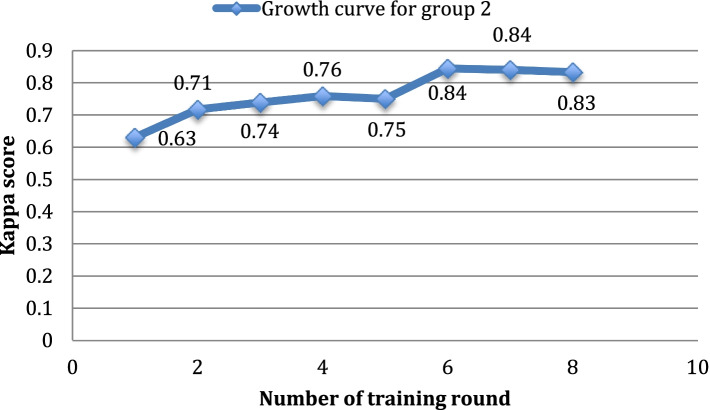
Growth curve of the average kappa scores for Group 2 (medical students). The abscissa is the number of training rounds, and the ordinate is the Kappa score

The 13 participants were divided into two groups. Group 1 consisted of junior ophthalmology residents who had basic knowledge of ophthalmology. Group 2 consisted of medical students who did not have any ophthalmology knowledge base. The average kappa score of each group was calculated separately. As shown in Table 2, after eight rounds of reading, the average kappa score of Group 1 was elevated from 0.71 to 0.76. The average kappa score of Group 2 was elevated from 0.63 to 0.84. Figures 2 and 3 show the growth curves according to the kappa scores of the two groups.

**Table 2:** Average kappa scores of the two groups

	Number of rounds							
	1	2	3	4	5	6	7	8
Group1	0.71	0.72	0.86	0.77	0.83	0.81	0.82	0.76
Group2	0.63	0.71	0.74	0.76	0.75	0.84	0.84	0.83

Group1: Junior ophthalmology residents who got basic knowledge of ophthalmology

Group2: Medical students who did not have any ophthalmology knowledge base

## Discussion

In recent years, AI technology based on classic machine learning (ML) or deep learning (DL) has been widely used in a variety of fundus disease screenings, including DR. Gulshan et al, who used the deep learning algorithm for the screening of DR and obtained extremely high sensitivity and specificity [10]. Takahashi et al. used a modified deep learning algorithm model for the screening and grading of DR, which can obtain grading results similar to those of ophthalmologists [20]. However, even if the application of AI technology in DR screening and grading has achieved very high accuracy, the final results can only be used as a diagnostic reference. Training junior ophthalmologists to grow rapidly and perform DR reading accurately is still an important part of ophthalmologist training. If junior ophthalmologists can master the DR reading method through centralized training quickly, it is not only conducive to the growth of ophthalmologists but also reserves the strength of physicians for labelling AI training sets. Therefore, it is of great significance to find an efficient DR reading training method. There is no previous discussion on the standard method of DR reading training, and there is no literature exploring the use of an AI reading label system for reading training and learning. In this study, the AI reading label system was used for the DR reading training of junior ophthalmology residents and medical students.

In this DR reading training, after 8 rounds of reading, the average kappa score of 13 participants increased from 0.67 in the first reading to 0.81 in the eighth reading. The average kappa score of the first four rounds was 0.77, indicating significant agreement, and the average kappa score of the last four rounds was 0.81, indicating that after training, the overall reading accuracy of the participants was significantly improved. The kappa score did not linearly increased each time, which may be because the difficulty level could not be completely consistent with the picture loaded at each time, resulting in the bias of the results.

In our previous studies, during the DR reading training of the AI dataset, we calculated the overall kappa scores for doctors of different seniorities. Seventeen attendings and six consultants in the fundus speciality read 20,503 fundus photographs, and the overall kappa scores were 0.67 for attendings and 0.71 for consultants [18, 21]. In our training, the overall kappa score was elevated from 0.67 to 0.81, with a higher score than the attendings and consultants, despite the trainees’ lower levels of professional training.

At the same time, the trainees were also divided into two groups for statistics. The first group consisted of junior ophthalmology residents with a certain basic knowledge of ophthalmology who also attended ophthalmology courses and participated in the clinical work of ophthalmology. The second group consisted of medical students who had not learned the basic knowledge of ophthalmology before the start of reading training and who had not participated in the courses and clinical work of ophthalmology after the start of reading. The initial kappa score of the two groups reflected the difference in the knowledge base of the two groups of readers, with an initial kappa score of 0.71 in Group 1 and 0.62 in Group 2, reflecting that the accuracy of the basic reading was higher in Group 1 than in Group 2. As the training progressed, the difference between the two groups gradually narrowed, and the kappa scores increased to 0.76 in Group 1 and 0.84 in Group 2 for the eighth reading, with a more significant increase in the medical student group. The average kappa score of the first four rounds was 0.77, and the average kappa score of the last four rounds was 0.81 in Group 1, 0.71 in the first four rounds and 0.82 in the last four rounds in Group 2, which also reflected that the gap in reading accuracy between the two groups was reduced. After reading training, even medical students without an ophthalmological knowledge base could be familiar with the law of DR reading and achieve a certain diagnostic accuracy.

The results of the harmonic mean of with/without DR, referral/non-referral DR, and severe/non-severe DR showed that the harmonic mean of with/without DR was the lowest, and the harmonic mean of referral/non-referral DR and severe/non-severe DR was relatively higher, which may be because it may not be precise for the presence or absence of microhemangioma based on fundus colour photography alone. The small microhemangioma in the picture may be confused with poor-quality artefacts at the time of photography, leading to incorrect conclusions. This also suggests that for the reading training, we should be cautious in selecting the fundus photographs used for the training, try to select the pictures with good quality, and eliminate the possible confounding factors caused by the poor quality of picture shooting.

This study also has some limitations. Since the original application of the reading label system used in the training was to train the AI deep learning model, which is not used for the physicians’ reading training, the system cannot immediately give the correct grading answer after labelling and needs to uniformly conduct the retrospective learning of picture grading after each label, which has an effect on the reading learning efficiency. Also, the number of people included in the training was small, and there may be some errors in the statistical mean. To make this system more conducive to reading training, the AI reading result prompt function can be added, and the gold standard is given after each round of picture labelling for comparison, which can increase training efficiency and strengthen the effect of reading training. The residents and medical students who participated in the training had different backgrounds in previous medical education and therefore had different foundations prior to training, which may have influenced the training outcomes to some extent. To reduce the bias caused by this factor, the participants’ basic ophthalmic knowledge needs to be examined, and they should be divided into different groups according to the results before training.

In conclusion, the use of an artificial intelligence DR reading label system can effectively improve the DR reading level of junior ophthalmologists and can achieve a certain reading accuracy in a short time with hundreds of images, which is a feasible reading training method.

## Data Availability

The datasets generated and/or analyzed during the current study are not publicly available due to limitations of ethical approval involving the patient data and anonymity but are available from the corresponding author on reasonable request.

## References

[1] Yau J, Rogers S, Kawasaki R (2012). Global prevalence and major risk factors of diabetic retinopathy. Diabetes Care.

[2] Gwatidzo S, Stewart WJ (2017). Diabetes mellitus medication use and catastrophic healthcare expenditure among adults aged 50+ years in China and India: results from the WHO study on global AGEing and adult health (SAGE). BMC Geriatr.

[3] Yang W, Lu J, Weng J (2010). Prevalence of diabetes among men and women in China. N Engl J Med.

[4] Wang L, Gao P, Zhang M (2017). Prevalence and ethnic pattern of diabetes and prediabetes in China in 2013. JAMA.

[5] Song P, Yu J, Chan KY (2018). Prevalence, risk factors and burden of diabetic retinopathy in China: a systematic review and meta-analysis. J Glob Health.

[6] Cheung N, Mitchell P, Wong T (2010). Diabetic retinopathy. Lancet.

[7] Wong T, Cheung C, Larsen M (2016). Diabetic retinopathy. Nat Rev Dis Primers.

[8] National Health Commission of the People’s Republic of China (2020). White paper on eye health in China.

[9] Gloucestershire Retinal Education Group. Introduction of “ Qualification: Certificate of Higher Education in Diabetic Retinopathy Screening”. https://www.gregcourses.com/certificate-of-higher-education-in-diabetic-retinopathy-screening.

[10] Gulshan V, Peng L, Coram M (2016). Development and validation of a deep learning algorithm for detection of diabetic retinopathy in retinal fundus photographs. JAMA.

[11] Abramoff M, Lou Y, Erginay A (2016). Improved automated detection of diabetic retinopathy on a publicly available dataset through integration of deep learning. Invest Ophthalmol Vis Sci.

[12] Ting D, Cheung C, Lim G (2017). Development and validation of a deep learning system for diabetic retinopathy and related eye diseases using retinal images from multiethnic populations with diabetes. JAMA.

[13] Gargeya R, Leng T (2017). Automated identification of diabetic retinopathy using deep learning. Ophthalmology.

[14] Abramoff M, Lavin P, Birch M (2018). Pivotal trial of an autonomous AI-based diagnostic system for detection of diabetic retinopathy in primary care offices. NPJ Digit Med.

[15] Gulshan V, Rajan R, Widner K (2019). Performance of a deep-learning algorithm vs manual grading for detecting diabetic retinopathy in India. JAMA Ophthalmol.

[16] Ruamviboonsuk P, Krause J, Chotcomwongse P (2019). Deep learning versus human graders for classifying diabetic retinopathy severity in a nationwide screening program. NPJ Digit Med.

[17] Li Z, Guo C, Nie D (2020). Development and evaluation of a deep learning system for screening retinal hemorrhage based on ultra-widefield fundus images. Transl Vis Sci Technol.

[18] Wang Y, Yu M, Hu B (2021). Deep learning-based detection and stage grading for optimising diagnosis of diabetic retinopathy. Diabetes Metab Res Rev.

[19] Wilkinson C, Ferris F, Klein R (2003). Proposed international clinical diabetic retinopathy and diabetic macular edema disease severity scales. Ophthalmology.

[20] Takahashi H, Tampo H, Arai Y (2017). Applying artificial intelligence to disease staging: deep learning for improved staging of diabetic retinopathy. Plos One.

[21] Zhang X, Li F, Li D, et al. Automated detection of severe diabetic retinopathy using deep learning method. Graefes Arch Clin Exp Ophthalmol. 2021. 10.1007/s—417-021-05402-x Online ahead of print. 10.1007/s00417-021-05402-x 34591173

